# Visual Pose Estimation of Rescue Unmanned Surface Vehicle From Unmanned Aerial System

**DOI:** 10.3389/frobt.2019.00042

**Published:** 2019-05-31

**Authors:** Jan Dufek, Robin Murphy

**Affiliations:** Department of Computer Science and Engineering, Texas A&M University, College Station, TX, United States

**Keywords:** visual pose estimation, visual localization, heterogenous multi-robot team, search and rescue robotics, field robotics, computer vision, marine robotics, aerial robotics

## Abstract

This article addresses the problem of how to visually estimate the pose of a rescue unmanned surface vehicle (USV) using an unmanned aerial system (UAS) in marine mass casualty events. A UAS visually navigating the USV can help solve problems with teleoperation and manpower requirements. The solution has to estimate full pose (both position and orientation) and has to work in an outdoor environment from oblique view angle (up to 85° from nadir) at large distances (180 m) in real-time (5 Hz) and assume both moving UAS (up to 22 m s^−1^) and moving object (up to 10 m s^−1^). None of the 58 reviewed studies satisfied all those requirements. This article presents two algorithms for visual position estimation using the object's hue (thresholding and histogramming) and four techniques for visual orientation estimation using the object's shape while satisfying those requirements. Four physical experiments were performed to validate the feasibility and compare the thresholding and histogramming algorithms. The histogramming had statistically significantly lower position estimation error compared to thresholding for all four trials (p-value ranged from ~0 to 8.23263 × 10^−29^), but it only had statistically significantly lower orientation estimation error for two of the trials (*p*-values 3.51852 × 10^−39^ and 1.32762 × 10^−46^). The mean position estimation error ranged from 7 to 43 px while the mean orientation estimation error ranged from 0.134 to 0.480 rad. The histogramming algorithm demonstrated feasibility for variations in environmental conditions and physical settings while requiring fewer parameters than thresholding. However, three problems were identified. The orientation estimation error was quite large for both algorithms, both algorithms required manual tuning before each trial, and both algorithms were not robust enough to recover from significant changes in illumination conditions. To reduce the orientation estimation error, inverse perspective warping will be necessary to reduce the perspective distortion. To eliminate the necessity for tuning and increase the robustness, a machine learning approach to pose estimation might ultimately be a better solution.

## 1. Introduction

**Figure 1:**
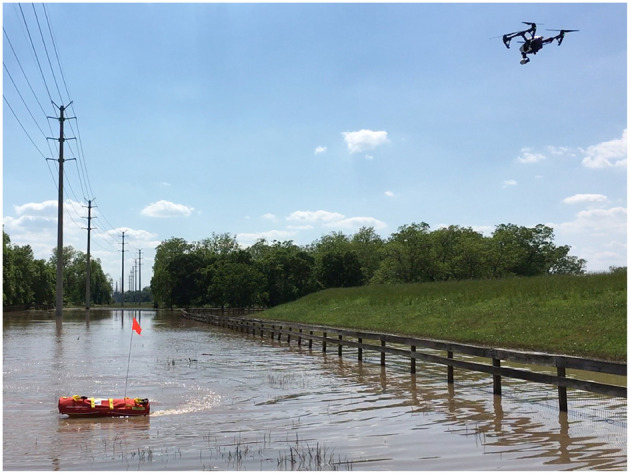
DJI Inspire 1 (UAS) assisting EMILY (USV) using visual feedback.

Using a UAS to visually navigate a rescue USV to victims can help responders during marine mass casualty events. First responders lack the manpower to effectively address marine mass casualty events during which it is not uncommon to have 80 victims in the water at the same time. While rescue USVs can help, teleoperation is problematic due to the lack of depth perception when USV is far away and the manpower requirements, and global positioning system (GPS) waypoint navigation is not precise enough as exact GPS coordinates of the victims are unknown and victims might drift with waves and currents. A UAS can visually navigate the USV to the victims by using visual feedback eliminating the need for teleoperation or GPS-based navigation as was shown in our previous work presented in Dufek et al. (2017), Karnan et al. (2017), Xiao et al. (2017), and Dufek and Murphy (2018). Such a heterogeneous multi-robot team of a UAS and a USV is illustrated in Figure 1.

**Figure 2:**
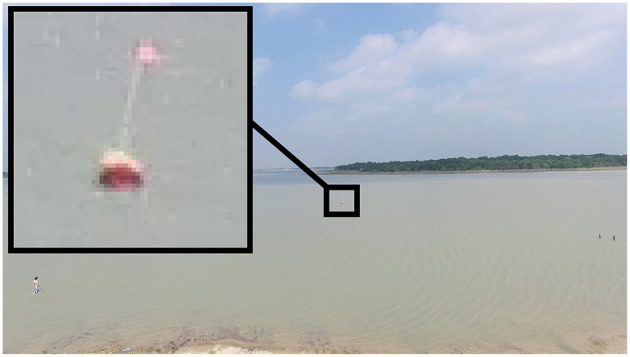
The spatial resolution of the object in the UAS view might be very low.

Visual navigation requires visual pose estimation of the USV which can be generalized to visual pose estimation of any fast moving object using UAS leading to the following problem statement: Visually estimate the pose of a fast maneuvering object using moving UAS, relative to UAS's image frame of reference, in an outdoor environment from an oblique view angle at large distances in real-time. In this article, a USV is taken as an instance of this object. The solution must satisfy working environment requirements, vehicular requirements, physical settings requirements, and desired output requirements. The working environment requirements are that the solution must work in an outdoor water environment where the rescues are being performed. Vehicular requirements are that the solution must work for moving UAS and moving USV. Small inexpensive UAS that would be used in rescue application (e.g., DJI Inspire 1) can move at speeds of up to 22 m s^−1^. Rescue- USVs (e.g., EMILY) can move at speeds of up to 22 m s^−1^ and make abrupt maneuvers with turn rate of up to 180 ° s^−1^. The physical settings requirements are that the solution must work at a large distance between UAS and USV and at an oblique view angle. The radius of marine mass casualty event rescue operations is typically 180 m. This distance causes the spatial resolution of the USV to be very low as illustrated in Figure 2 implicating that fiducial markers encoding full pose (e.g., AprilTag) would not be visible. The UAS might be operated above shore while USV is in the water causing the oblique view angle of up to 85° from nadir. The desired output requirements are that the output should be full pose (both position and orientation) and it should be updated in real-time (at least 5 Hz) to enable the visual navigation. While it might be beneficial to use multiple UAS as well as to estimate the pose of multiple objects simultaneously, for the sake of simplicity, it is assumed in this paper that there is only a single UAS and it is estimating the pose of a single object.

The contributions of this article are addressing a problem of how can a moving small UAS visually estimate the position and orientation of a fast maneuvering object, relative to UAS's image frame of reference, in an outdoor environment from an oblique view angle at large distances in real-time, that previous work failed to fully address, analyzing this problem on an example of a life-saving USV, proposing a method satisfying all the introduced requirements, and comparing two algorithms for solving this problem. First, this work addresses a problem defined above that none of the 58 reviewed studies fully covered. Second, it presents an analysis of this problem and justifies why some approaches would not work. Third, it presents a hue-based visual pose estimation method consisting of two algorithms for visual position estimation and four techniques for visual orientation estimation. Fourth, it compares two algorithms, thresholding and histogramming, for visual position estimation combined with one technique for orientation estimation in four physical experiments.

The rest of this article is organized as follows. Section 2 discusses related work in visual pose estimation of an object from UAS and identifies gaps and opportunities in research, most notably the lack of solution satisfying all the requirements discussed above. Section 3 presents two algorithms for position estimation (thresholding and histogramming) and four shape analysis techniques for orientation estimation. Section 4 details implementation of those algorithms on a laptop using C++ and OpenCV and describes platforms used for the implementation. Section 5 introduces experimental methodology to determine pose estimation error relative to manually annotated ground truth and presents results from four physical trials comparing thresholding and histogramming. Section 6 discusses reasons histogramming algorithm is better than thresholding (it can better model the object under local illumination conditions), points out problems with the proposed algorithms (particularly large orientation estimation error, necessity of tuning, and low robustness to significant illumination changes), examines how the experiments could have been improved (most notably testing for robustness in addition to precision), and indicates machine learning would be suitable to explore for future work. Finally, section 7 summarizes the article.

## 2. Related Work

Fifty-eight studies from the robotics and computer vision scientific literature were reviewed. While all the eight requirements introduced in section 1 were partially addressed by those studies, none satisfied all the requirements combined. The studies were classified to seven categories depending on what was the object for which the visual pose estimation using UAS was done: UGV (27 studies), car (9 studies), person (7 studies), landing platform (7 studies), standalone fiducial marker (3 studies), another UAS (2 studies), and other objects (3 studies). There were no studies on visual pose estimation of USV from UAS. All the categories except UGV category were limited in scope to only those studies where the visual pose estimation was done outdoors.

Each study was evaluated using eight criteria to validate if the particular study satisfied the requirements introduced in section 1. To validate the working environment requirements, one criterion was evaluated: (1) Were the experiments performed indoors or outdoors? To validate the vehicular requirements, two criteria were evaluated: (1) Was the UAS static or moving? (2) Was the object static or moving? To validate physical settings requirements, three criteria were evaluated: (1) Was the distance between UAS and the object short or long (distance is considered short for the purpose of this review if less than or equal 30 m)? (2) Was a fiducial marker used or not? (3) Was the view from UAS nadir or oblique? To validate desired output requirements, two criteria were evaluated: (1) Was just a position estimated or full pose (both position and orientation)? (2) Was the output generated real-time (i.e., updating at least at 5 Hz)? Table 1 lists all the studies and for each, it shows which criteria were satisfied.

**Table 1:** The requirements violations for the 58 reviewed studies.

**References**	**Object**	**Outdoors**	**Moving UAS**	**Moving object**	**Oblique view**	**Large distance**	**Estimated distance**	**No fiducials**	**Full pose**	**Real-time**	**Total violations**
Dixon et al., 2001	UGV	✕	✕	✓	✕	✕		✕	✓	✓	6
Rao et al., 2003	UGV	✕	✕	✓	✓	✕		✕	✓	✓	5
Rao et al., 2004	UGV	✕	✕	✓	✓	✕		✕	✓	✓	5
Rao et al., 2005	UGV	✕	✕	✓	✓	✕		✕	✓	✓	5
Rao et al., 2006	UGV	✕	✕	✓	✓	✕		✕	✓	✓	5
Cognetti et al., 2014	UGV	✕	✕	✓	✕	✕		✕	✓	✓	6
Gao et al., 2014	UGV	✕	✓	✓	✓	✕		✓	✕	✓	3
Aranda et al., 2015	UGV	✕	✕	✓	✕	✕		✕	✓	✓	6
Harik et al., 2015a	UGV	✕	✕	✓	✕	✕		✕	✓	✓	6
Harik et al., 2015b	UGV	✕	✕	✓	✕	✕		✕	✓	✓	6
Hausman et al., 2015	UGV	✕	✓	✓	✕	✕		✕	✓	✓	4
Laiacker et al., 2015	UGV	✓	✓	✕	✕	✕	10 m	✕	✓	✓	4
Rosa et al., 2015	UGV	✕	✕	✓	✕	✕		✕	✓	✓	6
Byun et al., 2016	UGV	✕	✓	✕	✕	✕		✕	✕	✓	6
Cantelli et al., 2016	UGV	✓	✓	✓	✕	✕	5 m	✕	✓	✓	3
Chen et al., 2016	UGV	✕	✓	✓	✕	✕		✓	✕	✓	4
Harik et al., 2016	UGV	✕	✓	✓	✕	✕		✕	✓	✓	4
Hausman et al., 2016	UGV	✕	✓	✓	✕	✕		✕	✓	✓	4
Santana et al., 2016	UGV	✓	✓	✓	✕	✕	1 m	✕	✓	✓	3
Wang et al., 2016	UGV	✕	✓	✓	✕	✕		✕	✓	✓	4
Araar et al., 2017	UGV	✕	✓	✕	✕	✕		✕	✓	✓	5
Battiato et al., 2017	UGV	✓	✓	✕	✕	✕	10 m	✕	✕	✓	5
Harik et al., 2017	UGV	✕	✓	✓	✕	✕		✕	✓	✓	4
Hoang et al., 2017	UGV	✕	✓	✓	✕	✕		✕	✓	✓	4
Wang et al., 2017	UGV	✕	✓	✓	✕	✕		✕	✓	✓	4
Gomez-Avila et al., 2018	UGV	✕	✓	✓	✕	✕		✕	✓	✓	4
Harikumar et al., 2018	UGV	✓	✓	✓	✕	✕	10 m	✕	✓	✓	3
Siam et al., 2012	Car	✓	✓	✓	✓	✓	70 m	✓	✕	✓	1
Siam and ElHelw, 2012	Car	✓	✓	✓	✓	✓	70 m	✓	✕	✓	1
van Eekeren et al., 2015	Car	✓	✓	✓	✓	✓	500 m	✓	✕	✓	1
Ma et al., 2016	Car	✓	✕	✓	✕	✓	70 m	✓	✕	✕	4
Watanabe et al., 2016	Car	✓	✓	✓	✕	✕	30 m	✓	✓	✓	2
Askar et al., 2017	Car	✓	✓	✓	✓	✓	70 m	✓	✕	✓	1
Chen et al., 2017	Car	✓	✕	✓	✕	✓	70 m	✓	✕	✕	4
Kim et al., 2017	Car	✓	✕	✓	✓	✓	50 m	✓	✕	✕	3
Kaufmann et al., 2018	Car	✓	✕	✓	✓	✓	100 m	✓	✕	✓	2
Lim and Sinha, 2015	Person	✓	✓	✓	✓	✕	10 m	✓	✕	✓	2
Mendonça et al., 2016	Person	✓	✓	✕	✕	✕	10 m	✓	✕	✓	4
Bian et al., 2016	Person	✓	✓	✓	✓	✕	10 m	✓	✕	✓	2
Monajjemi et al., 2016	Person	✓	✓	✕	✓	✕	30 m	✓	✕	✓	3
Cheng et al., 2017	Person	✓	✓	✓	✓	✕	5 m	✓	✕	✓	2
Lee et al., 2018	Person	✓	✓	✓	✓	✕	30 m	✓	✕	✓	2
Liu et al., 2018	Person	✓	✓	✓	✓	✕	14 m	✓	✕	✓	2
Medeiros et al., 2015	Landing platform	✓	✓	✕	✕	✕	10 m	✕	✓	✓	4
Kim et al., 2016	Landing Platform	✓	✓	✓	✕	✕	10 m	✕	✕	✓	4
Lee et al., 2016	Landing platform	✓	✓	✓	✕	✕	10 m	✕	✕	✓	4
Cabrera-Ponce and Martinez-Carranza, 2017	Landing platform	✓	✓	✕	✕	✕	5 m	✕	✕	✓	5
Collins et al., 2017	Landing platform	✓	✓	✕	✕	✕	22 m	✕	✓	✓	4
Junaid et al., 2017	Landing platform	✓	✓	✕	✕	✕	5 m	✕	✕	✓	5
Patruno et al., 2018	Landing platform	✓	✓	✕	✕	✕	5 m	✕	✓	✓	4
Feng et al., 2007	Standalone Fiducial marker	✓	✕	✕	✕	✕	10 m	✕	✕	✓	6
Cho et al., 2016	Standalone Fiducial marker	✓	✓	✕	✓	✓	200 m	✕	✕	✓	3
Hinas et al., 2017	Standalone Fiducial marker	✓	✓	✕	✕	✕	30 m	✕	✕	✓	5
Liu and Feng, 2018	UAS	✓	✓	✓	✓	✕	30 m	✓	✕	✓	2
Wang et al., 2018	UAS	✓	✕	✕	✓	✕	5 m	✓	✓	✕	4
Máthé et al., 2016	Railway semaphore	✓	✓	✕	✓	✕	5 m	✓	✕	✓	3
Koo et al., 2017	Jellyfish	✓	✕	✕	✕	✕	10 m	✓	✕	✓	5
Liu et al., 2017	General objects	✓	✓	✕	✕	✕	15 m	✓	✕	✓	4
Violationsperrequirement		22	17	18	37	49		35	30	4	

*The distance between the UAS and the object was considered large if more than 30 m. The numerical distance is only listed for outdoor studies and it was estimated from particular study's figures if not explicitly reported. A study was considered real-time if the update rate was more than 5 Hz. The last column lists the total number of requirements violations for a particular study. The last row lists the total number of studies that violated a particular requirement. The studies are grouped by the object for which the visual pose estimation was done. The groups are ordered by group size. Inside a single group, studies are ordered by year and then by first author last name. Green tick indicates the corresponding study satisfies corresponding requirement and red cross otherwise*.

A UAS has been used to visually estimate the pose of UGV in 27 studies relying on fiducial markers and the nadir view or only tested indoors at a short distance. Majority of those studies (20 out of 27) relied on a fiducial marker mounted on the UGV and assumed the nadir view (Dixon et al., 2001; Cognetti et al., 2014; Aranda et al., 2015; Harik et al., 2015a,b, 2016, 2017; Hausman et al., 2015, 2016; Laiacker et al., 2015; Rosa et al., 2015; Byun et al., 2016; Cantelli et al., 2016; Santana et al., 2016; Wang et al., 2016, 2017; Araar et al., 2017; Battiato et al., 2017; Gomez-Avila et al., 2018; Harikumar et al., 2018). Four studies assumed an oblique view, but still relied on fiducial markers mounted on the UGV (Rao et al., 2003, 2004, 2005, 2006). Only three studies estimated pose of UGV without any fiducial markers. Gao et al. (2014) proposed a method to track UGV from UAS, but only the position was estimated (not orientation) and the method was only tested indoors at a short distance. Chen et al. (2016) also estimated position only (no orientation) of UGV with UAS flying directly above at a short distance looking nadir in an indoor experiment. Hoang et al. (2017) proposed a method to track a UGV, but it was tested only indoors at a short distance (1.5 m).

A UAS has been used to visually estimate the pose of cars in nine studies estimating position only or requiring the nadir view (Siam and ElHelw, 2012; Siam et al., 2012; van Eekeren et al., 2015; Ma et al., 2016; Watanabe et al., 2016; Askar et al., 2017; Chen et al., 2017; Kim et al., 2017; Kaufmann et al., 2018). All those studies except Watanabe et al. (2016) only estimated the position and not orientation. The distance between the UAS and the car/cars was relatively long, but at the same time, cars are large objects so the object's spatial resolution was still relatively high. Four of those studies (Ma et al., 2016; Chen et al., 2017; Kim et al., 2017; Kaufmann et al., 2018) used static UAS and the proposed methods required known ground reference points in the environment. For three studies (Ma et al., 2016; Chen et al., 2017; Kim et al., 2017), pedestrians were tracked as well, but the methods were not real-time. Ma et al. (2016) and Chen et al. (2017) assumed the nadir view and constant altitude. For Watanabe et al. (2016), orientation was estimated, but the nadir view was assumed and the distance between UAS and the car was short. Van Eekeren et al. (2015) used very high resolution images (116 Mpx) from high altitude. While the view was oblique, the very high altitude caused the view to appear very close to nadir. This method also required a 3D reconstruction to get the target's height and only worked with cars moving faster than 10 km h^−1^.

A UAS has been used to visually estimate the pose of a person in seven studies estimating position only on short distances (Lim and Sinha, 2015; Bian et al., 2016; Mendonça et al., 2016; Monajjemi et al., 2016; Cheng et al., 2017; Lee et al., 2018; Liu et al., 2018). In those studies, only the position was estimated (not orientation) and the person was close to the UAS. The method proposed in Lim and Sinha (2015) additionally required the person's height. For Mendonça et al. (2016) and Monajjemi et al. (2016), the target person was assumed to be static. For Mendonça et al. (2016), the nadir view from UAS was assumed.

A UAS has been used to visually estimate the pose of a landing platform during an autonomous landing of a UAS in seven studies relying on fiducial markers, the nadir view, and short distances (Medeiros et al., 2015; Kim et al., 2016; Lee et al., 2016; Cabrera-Ponce and Martinez-Carranza, 2017; Collins et al., 2017; Junaid et al., 2017; Patruno et al., 2018). All of those studies assumed the nadir view and used a fiducial marker on the landing platform. All the experiment were done on a short distance since for autonomous landing the visual pose estimation of a landing platform is only done in the final stages of landing. All the studies except two (Kim et al., 2016; Lee et al., 2016) used static landing platform. All studies except three (Medeiros et al., 2015; Collins et al., 2017; Patruno et al., 2018) estimated only position and not orientation.

A UAS has been used to visually estimate the pose of a standalone fiducial marker in three studies assuming static object and estimating position only (Feng et al., 2007; Cho et al., 2016; Hinas et al., 2017). Two studies (Feng et al., 2007; Hinas et al., 2017) assumed the nadir view and were tested on a short distance. In addition, Feng et al. (2007) assumed a static UAS. A UAS has been used to visually estimate the pose of another UAS in two studies estimating position only or not running in real-time and tested only on a short distance (Liu and Feng, 2018; Wang et al., 2018). Both methods did pose estimation on a short distance. The method proposed by Wang et al. (2018) was not real-time and both UASs were static. Liu and Feng (2018) estimated the position only and not the orientation.

Finally, a UAS has been used to visually estimate the pose of three kinds of other objects in three studies all assuming static object, estimating position only, and testing only on a short distance. Máthé et al. (2016) estimated pose of railway semaphores, Koo et al. (2017) estimated pose of jellyfish in the water, and Liu et al. (2017) estimated pose of general objects. All those studies estimated only position (not orientation) on a short distance and assumed static objects. All except Máthé et al. (2016) assumed the nadir view. All except Koo et al. (2017) assumed a constant distance between UAS and objects, making specific assumptions about object sizes. Koo et al. (2017) also assumed static UAS. Máthé et al. (2016) had view fixed in a single horizontal plane looking forward.

None of the 58 reviewed studies satisfied all the requirements introduced in section 1. The 58 studies had one or more of the requirement violations: indoor experiments only (22 studies), static UAS (17 studies), static object (18 studies), the nadir view only (37 studies), short distance less than 30 m between UAS and the object (49 studies), fiducial markers (35 studies), not estimating full pose including both position and orientation (30 studies), or not real-time with update at least 5 Hz (4 studies).

## 3. Approach

The review of the literature indicates that a method is needed for visual pose estimation, that would satisfy all the requirements: working outdoors, moving UAS, moving object, oblique view angle, large distance more than 30 m, no fiducial markers, estimating full pose including both position and orientation, and real-time update rate of more than 5 Hz. The approach taken in this article is based on the object's blob hue that is relatively invariant compared to the object's blob brightness, convexity, size, inertia ratio, features, or motion. Two algorithms for position estimation are presented. The first algorithm, thresholding, consists of seven steps applied for each video frame: blur, HSV conversion, value histogram equalization, thresholding, erosion, dilation, and contours detection. The second algorithm, histogramming, first takes user input to construct a hue histogram model of the object and then applies six steps on each video frame: blur, HSV conversion, value histogram equalization, hue histogram backprojection, thresholding on saturation and value, and CamShift algorithm for finding and tracking objects. Shape analysis is used for orientation estimation assuming the major axis of the object's blob indicates the object's blob heading. A total of four techniques for estimation of the principal axis of a blob are examined: line fitting, rectangle fitting, principal component analysis (PCA), and ellipse fitting.

**Figure 3:**
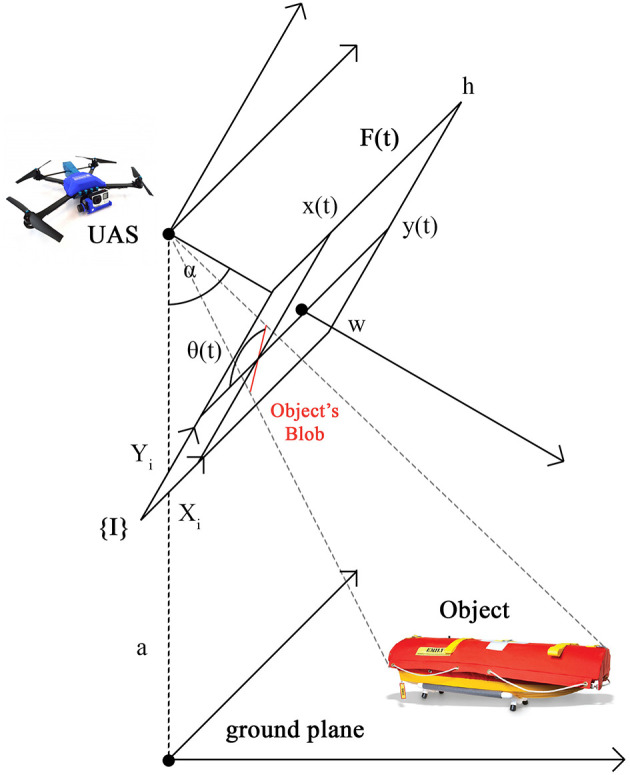
The physical configuration of the problem. {*I*} is UAS 2D image coordinate system with axes *X*_*i*_ and *Y*_*i*_. *F*(*t*) is UAS video frame at time *t* with resolution *w* × *h*. *a* is UAS altitude above ground level. α is UAS camera angle from nadir. (*x*(*t*), *y*(*t*)) is the object's blob position, that is the centroid of the object's blob in the coordinate frame {*I*}. θ(*t*) is the object's blob orientation, that is the angle between the object's blob heading and horizontal line (axis *X*_*i*_) in the coordinate frame {*I*}.

The problem of visually estimating the pose of an object can be decomposed into two parts, position estimation and orientation estimation, where input for both are video frames from the UAS. The input is a sequence of *n* video frames *F*(*t*), *t* ∈ [1..*n*] taken from a UAS with resolution *w* × *h, w* ∈ ℕ, *h* ∈ ℕ. The UAS is flying at altitude *a* above ground level and the UAS camera angle is α from nadir. A frame is defined as *F*(*t*) = ((*R, G, B*)_*u, v*_), *u* ∈ [1..*w*], *v* ∈ [1..*h*], *R, G, B* ∈ [0..255]. For the visual position estimation, the output is the coordinates of the centroid of the object's blob, **x**(*t*) = (*x*(*t*), *y*(*t*)), in UAS 2D image coordinate system, {*I*}, at time *t*. For the visual orientation estimation, the output is an angle between the object's blob heading and horizontal line, θ(*t*), in UAS 2D image coordinate system, {*I*}, at time *t*. Then, the pose at time *t* is a tuple (**x**(*t*), θ(*t*)). The physical configuration of the problem is depicted in Figure 3.

The object's blob has to be first identified in the video frame, however, identification using the object's blob brightness, convexity, size, inertia ratio, features, or motion is problematic as those attributes are not invariant; however, object's blob hue is relatively invariant and can be exploited. The object is represented as a blob of pixels in the UAS video frame *F*(*t*). To reliably identify this blob, some invariant property of this blob relative to changing *t* has to be identified.

**Figure 4:**
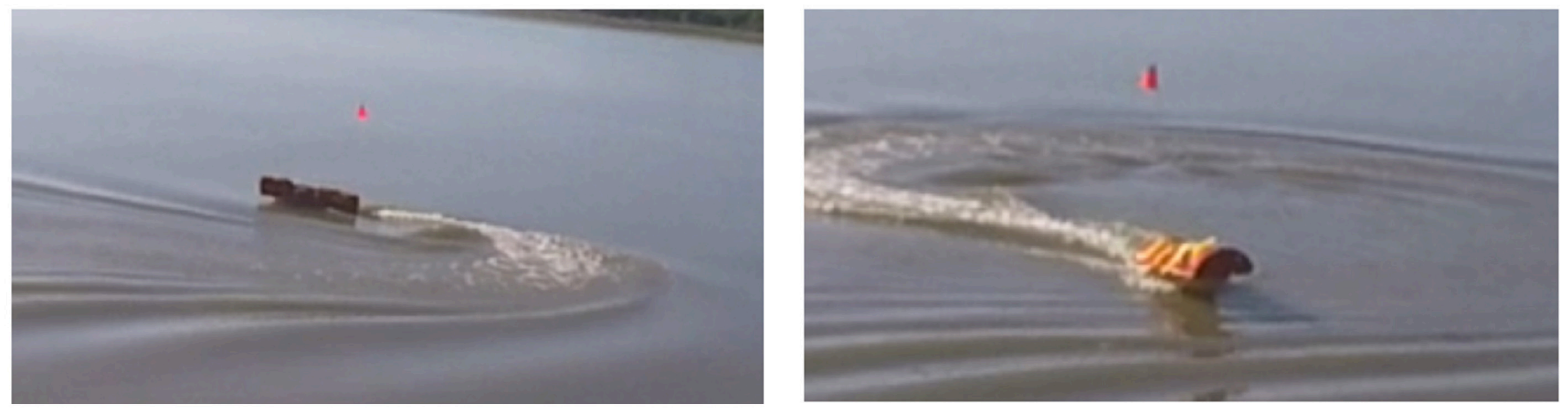
Object's blob brightness may vary significantly in the video frames *F*(*t*) with varying *t* because of the outdoor environmental conditions.

**Figure 5:**
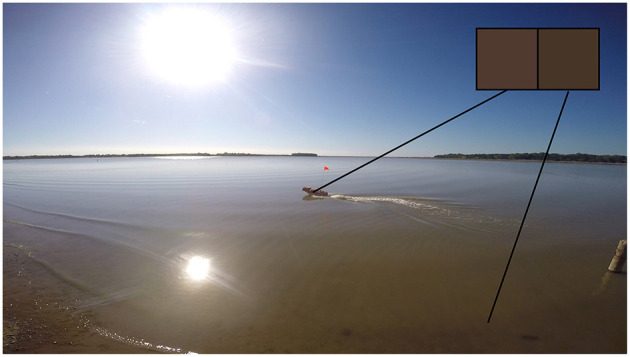
The illumination conditions may change with the relative position of the UAS, object, and sun.

The brightness of the blob representing the object in the video frames *F*(*t*) is not invariant in *t* because of the outdoor environmental conditions, particularly weather and sun. The brightness may change frequently depending on the weather and the relative pose of the object, UAS, and the sun. An example of weather affecting brightness are clouds that might temporarily cast shadows on the object. An example of the relative pose influence on the brightness is when the object that faces the UAS with its non-illuminated side changes its pose in a way that it now faces the UAS with its sun illuminated side as shown in Figure 4. In the figure, the brightness of a USV's blob changes as the USV completes a turn. The USV first displays its non-illuminated side making its blob look dark (left) and then after completing the turn, it displays the sun-illuminated side making its blob look bright (right). Another example is when the sun is in the field of view of the UAS's camera causing the white balance distortion in the video frames as can be seen in Figure 5. Consequently, the USV's color is very similar to the color of the surrounding water.

The convexity and concavity of the blob representing the object in the video frames *F*(*t*) is not invariant in *t* because of the outdoor environmental conditions, particularly water occlusions and shadows. The object might be temporarily partially occluded by water (waves or wake) or shadows causing the convexity and concavity of the blob to change over time.

**Figure 6:**
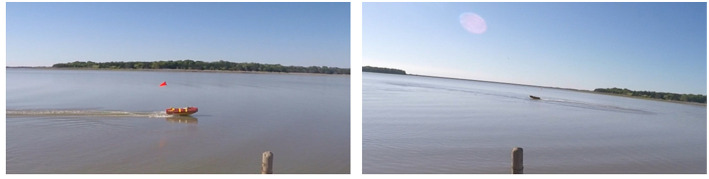
Object's blob size may differ significantly in the video frames *F*(*t*) with varying *t* because of the oblique view angle.

The size of the blob representing the object in the video frames *F*(*t*) is not invariant in *t* due to the oblique view angle α causing the distance from the UAS to change. The apparent size of the object in the image may change frequently depending on the distance of the object from the UAS and the relative pose of the object to the UAS. If the object moves away from the UAS, its size will decrease and vice versa as can be seen in Figure 6. In this figure, the relative size of the USV on the left is much larger than on the right because the USV is closer to the camera. Therefore, a priori assumptions about the object's blob size in video frames cannot be made.

**Figure 7:**
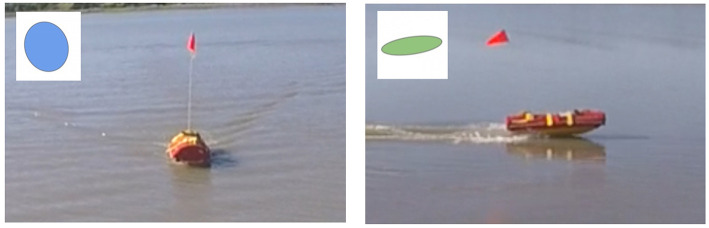
The ratio of the minimum inertia to maximum inertia of the object's blob may differ significantly in the video frames *F*(*t*) with varying *t* because of the oblique view angle.

The ratio of the minimum inertia to maximum inertia of the blob representing the object in the video frames *F*(*t*) is not invariant in *t* due to the oblique view angle α causing the object's shape to change. The inertia ratio of the blob depends on the relative orientation of the object relative to the UAS. Taking a cylindrical object such as a USV as an example, if such object is facing toward the UAS, its blob will be circular (inertia ratio will be high), however, If it is turned sideways, its blob will be elliptical (inertia ratio will be low) as shown in Figure 7. On the left side of the figure, USV has a high inertia ratio comparing to the low inertia ratio on the right because it faces the camera.

Visual features of the blob representing the object in the video frames *F*(*t*) are not invariant in *t* due to the potentially large distance between the object and the UAS. The object's blob might start relatively close to the UAS and then move very far (up to 180 m) causing the features to be lost with low spatial resolution. In the extreme case, the object's blob might be featureless due to the large distance between the object and the UAS causing the spatial resolution might be very low as illustrated in Figure 2. The figure shows DJI Phantom 3 Professional video feed with resolution 2,132 × 1,200 px with USV being approximately 100 m away. As can be seen in the close-up view, the spatial resolution of USV is very low (about 10 cm px^−1^). The USV appears as a circle with the radius of only 5 px. The USV's image blob area is only about 80 px taking only 0.003 % of the image.

**Figure 8:**
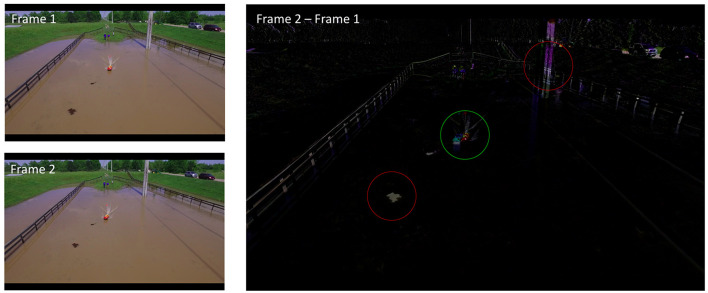
Moving UAS causes the stationary background to appear moving in the video frames.

The motion of the blob representing the object in the video frames *F*(*t*) is not invariant in *t* due to the motion of the UAS. The UAS motion causes two problems. First, the motion of the object in the real world does not correspond to the motion of the object's blob in the video frames. For example, if both the object and the UAS are moving at the same speed in the same direction, the object's blob will be static in the video frames. Second, the UAS motion causes the background to move as well as shown in Figure 8. In the figure, two adjacent video frames from a moving UAS (on the left) were subtracted to reveal anything that moved in between those frames (on the right). It can be seen, that the USV (green circle) moving in the real world was not the only object that moved between the frames. The electric power pole (top red circle), the debris in the water (bottom red circle), the fence, and the parked cars all moved between the frames as well even though they were stationary in the real world. This background movement is not uniform due to the parallax effect. The parts of the background closer to the UAS will appear to move faster than the parts of the background further away.

The hue of the blob representing the object in the video frames *F*(*t*), unlike the brightness, convexity, size, inertia ratio, features, or motion, is relatively invariant in *t* and can be exploited. This assumes the object has a combination of colors that is unique in the UAS view. Those colors can then be exploited in identifying the object's blob. While the saturation and value of a particular color might change with illumination, hue should be relatively invariant. This hue-based approach satisfies all the requirements discussed in section 1. The assumption about the uniqueness of colors is reasonable for rescue USVs and might be reasonable for other objects as well.

**Figure 9:**
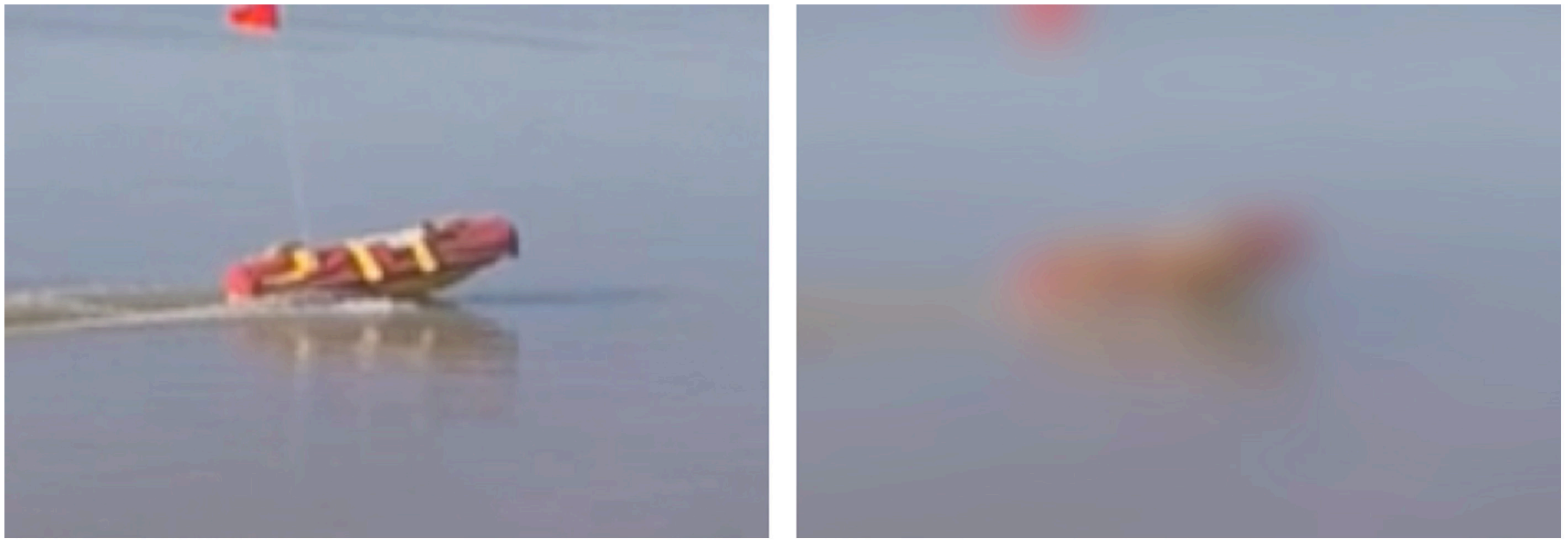
The color of the object might not be uniform which can be alleviated by Gaussian blur convolution filter.

There are two challenges with using the hue of the blob representing the object for the identification, non-uniformity of the object's color and difficulty to specify particular hue. First, the color of the object might not be uniform. For example, EMILY, a rescue USV, is mostly red but has many non-red parts on its surface. The object's color non-uniformity might cause that the object is represented by multiple blobs of different colors in the video frames. This might be a problem because the smaller color blobs might disappear and appear with changing spatial resolution as the object moves away or toward the UAS. This problem can be alleviated by using Gaussian blur convolution filter to diffuse the color of the smaller color blobs into the bigger color blobs as illustrated in Figure 9. The USV on the left side of the figure is composed of multiple color blobs. After the application of Gaussian blur convolution filter, smaller blobs are blended into bigger blobs as can be seen on the right side of the figure. The Gaussian filter can be applied by convolving the original image with a Gaussian kernel as follows:

$$g\left(i,j\right)=\underset{k,l}{\sum }f\left(i+k,j+l\right)G\left(k,l\right)$$

Where *g* is the new image, *f* is the original image and *G*(*k, l*) is the Gaussian kernel defined as follows:

$$G\left(k,l\right)=\frac{1}{2\pi \sigma^{2}}e^{-\frac{k^{2}+l^{2}}{2\sigma^{2}}}$$

Where *k* is the distance from the origin in the horizontal axis, *l* is the distance from the origin in the vertical axis, and σ is the standard deviation of the Gaussian distribution.

The second challenge is that specific hue is difficult to define in conventional RGB color space used by regular UAS visual cameras. The RGB values might be different in all three coordinates and still represent the same hue. It is also problematic to recognize changes in the saturation and intensity of a particular hue. An object with the specific hue under the varying intensity of illumination will have different saturation and value leading to different RGB values. The solution to this problem is the conversion to HSV color space. The HSV color space specifies a color in terms of hue, saturation, and value. In this color space, a hue can be specified independently from its saturation and value, which both change with illumination changes. Given an RGB image, the HSV can be calculated as follows:

V=max(R,G,B)

$$S=\{\begin{array}{ll} \frac{V-\min(R,G,B)}{V} & \text{if }V\neq 0\\ 0 & \text{otherwise} \end{array}$$

$$H=\{\begin{array}{cc} \frac{60(G-B)}{V-\min(R,G,B)} & \text{if }V=R\\ \frac{120+60(B-R)}{V-\min(R,G,B)} & \text{if }V=G\\ \frac{240+60(R-G)}{V-\min(R,G,B)} & \text{if }V=B \end{array}$$

Where *R* is the red channel, *G* is the green channel, *B* is the blue channel, *H* is hue, *S* is saturation, and *V* is value. If *H* < 0, then *H* is adjusted to *H* = *H* + 360.

### 3.1. Two Position Estimation Algorithms

Two algorithms for position estimation are introduced. The first is thresholding based on HSV thresholds and contour detection. The second is histogramming based on hue histogram model of the object, its backprojection, and CamShift tracking algorithm. Input for both algorithms are the video frames *F*(*t*) from UAS and the output is the object's position **x**.

While thresholding and CamShift have been used for vision-based object tracking before, the basic sequence of steps has been extended to address the challenges discussed at the beginning of section 3 and requirements discussed in section 1. The basic steps for thresholding application to object tracking are the actual thresholding and contours detection. Some studies also used erosion and dilation after thresholding step (Rosin and Ellis, 1995; Intille et al., 1997; Rosin, 2009; Seenouvong et al., 2016). The basic steps for object tracking based on CamShift are the conversation to HSV color space, hue histogram backprojection, and CamShift itself (Chen et al., 2012; Kamate and Yilmazer, 2015). The additional steps discussed below and their order are proposed by the authors specifically for the problem in hand.

#### 3.1.1. Thresholding

**Figure 10:**
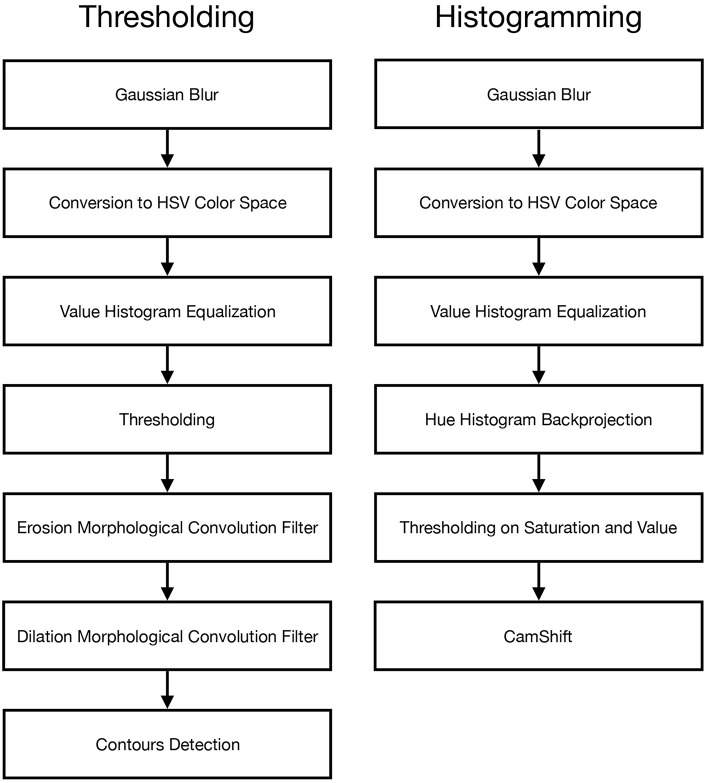
Flowcharts for the thresholding and histogramming algorithms.

The first, more naive, algorithm is based on thresholding of HSV values. Each input video frame *F*(*t*) from UAS goes through a series of seven steps: blur, HSV conversion, value histogram equalization, thresholding, erosion, dilation, and contours detection. This processed is schematized in the left flowchart in Figure 10. The final result of this series is the estimated **x** taken as the centroid of the largest area contour found in the last step.

The first and second step is Gaussian blur convolution filter and conversion to HSV color space as discussed above. The third step is histogram equalization on value plane of HSV to increase the global contrast in the video frame. The resulting image can be defined as:

$$g\left(i,j\right)=H^{\prime }\left(f\left(i,j\right)\right)$$

Where *g* is the new image, *f* is the original image, and *H*′ is the integral of the normalized histogram *H* of the image *f*. *H*′ can be calculated as follows:

$$H^{\prime }\left(l\right)=\underset{0\leq k<l}{\sum }H\left(k\right)$$

**Figure 11:**
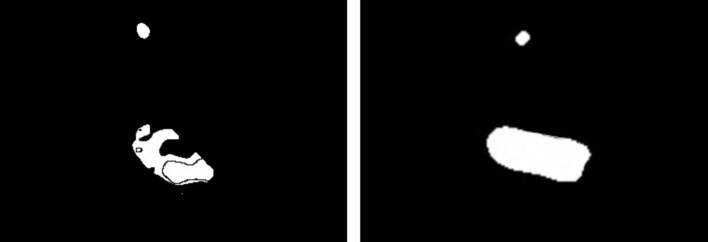
Erosion and dilation filters can be applied to the binary threshold map **(Left)** to filter out noise, smooth shapes, and fill-in holes **(Right)**.

The fourth step is the application of binary thresholding to identify pixels lying within the specified range of HSV values. The result is a binary threshold map where true signifies that the corresponding pixel is within the specified range of HSV values, and false otherwise. An example of a binary threshold map can be seen on the left side of Figure 11. The binary threshold map is created as follows:

$$g\left(i,j\right)=T_{min}\leq f\left(i,j\right)\leq T_{max}$$

Where *g* is the resulting binary threshold map, *f* is the original image, *T*_*min*_ is the lower range, and *T*_*max*_ is the upper range.

The fifth step is the application of erosion morphological convolution filter to filter out noise. The binary threshold map inherently contains noise. The erosion sets to false a specified number of true pixels that border with false pixels in the binary threshold map by using minimum function in the convolution kernel. It ultimately deletes very small dispersed clusters of true pixels and leaves just larger clusters. The resulting image can be defined as:

$$g\left(i,j\right)=\underset{\left\{\left(l,k\right)|\lambda \left(l,k\right)\neq 0\right\}}{\min}f\left(i+k,j+l\right)$$

Where *g* is the new image, *f* is the original image, and λ is the structuring element defining the shape of the neighborhood defined as:

$$\lambda (i,j)=\{\begin{array}{ll} 1 & \text{if }(i,j)\text{ is in the neighborhood}\\ 0 & \text{otherwise} \end{array}$$

The sixth step is the application of dilation morphological convolution to amplify the remaining clusters, smooth shapes, and fill-in possible holes in the clusters. The dilation sets to true a specified number of false pixels that border with true pixels in the binary threshold map by using maximum function in the convolution kernel. The holes might occur due to non-uniformity of the object's surface and illumination effects in the environment. An example of a binary threshold map after the application of erosion and dilation can be seen on the right side of Figure 11. The resulting image can be defined as:

$$g\left(i,j\right)=\underset{\left\{\left(l,k\right)|\lambda \left(l,k\right)\neq 0\right\}}{\max}f\left(i+k,j+l\right)$$

Where *g* is the new image, *f* is the original image, and λ is the structuring element defining the shape of the neighborhood.

The final, seventh, step is determining contours in the binary threshold map to identify blobs using the method proposed in Suzuki and Be (1985). Contours are boundaries of self-contained clusters of true pixels in the binary threshold map. The contours are found by following borders between true and false pixels in the binary map. Each contour then represents a single blob. In the case there is more than a single blob, the one with the largest area is selected. Then the **x** is computed as the centroid of this largest blob.

#### 3.1.2. Histogramming

The second algorithm is based on the construction of the hue histogram model and CamShift algorithm (Bradski, 1998). The main idea is to use a hue histogram instead of a simple hue range and then apply CamShift on backprojection of this hue histogram. This algorithm constructs hue histogram at the beginning using user's input and then takes each video frame through a series of six steps: blur, HSV conversion, value histogram equalization, hue histogram backprojection, thresholding on saturation and value, and CamShift algorithm for finding and tracking objects. This processed is schematized in the right flowchart in Figure 10.

Before the visual position estimation can begin, a hue histogram model of the object must be constructed using a user's selection of the object. This procedure is done only once in the beginning and not for every video frame. User input is required to select the object in the video frame. A hue histogram model is then constructed for the selected area. The hue histogram divides the entire hue range into a predefined number of non-overlapping bins of the same size. Each bin corresponds to a specific range of hue values. The model is constructed by counting for each bin how many pixels in the selected area are within the hue range of that bin. The histogram is only built for hue because the saturation and value of the object may change with variations in illumination. After the hue histogram is constructed, each input video frame *F*(*t*) from UAS goes through a series of six steps. The final result of this series is the estimated **x** taken as the centroid of the area found by CamShift.

**Figure 12:**
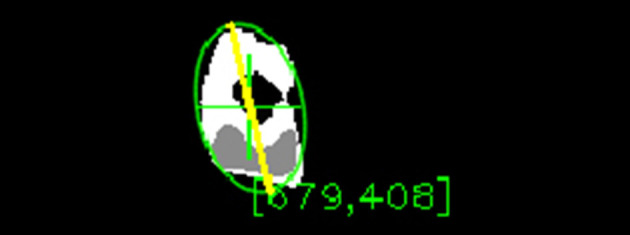
The backprojection of the object's histogram model and subsequent application of CamShift.

The first three steps are the same as for the thresholding algorithm presented in section 3.1.1: Gaussian blur convolution filter, conversion to HSV color space, and value histogram equalization. The fourth step is the calculation of the hue histogram backprojection to identify how well each pixel in *F*(*t*) fits the histogram distribution using the method proposed in Swain and Ballard (1990). The result is a greyscale map of the same dimensions as *F*(*t*), where each pixel's value signifies how much this pixel's hue is represented in the histogram as shown in Figure 12. This is calculated from the histogram by finding how many samples are in the histogram bin corresponding to the pixel's hue. The number of the samples is then normalized by dividing it by the number of samples in the bin with the most samples. The fifth step is the computation of binary threshold on saturation and value in order to filter out the pixels with low saturation and value. This binary threshold is computed on the original video frame independently of the backprojection. Only the pixels lying in the predefined range of saturation and value are set to true in this binary threshold map. This map is then joined with the backprojection map using logical and operation. This sets all the pixels that are false in the binary threshold map to 0 in the backprojection map while keeping everything else intact. This effectively filters out the pixels that do not belong to the specified saturation and value range from the backprojection. The sixth step is the application of CamShift algorithm (Bradski, 1998) for finding and tracking objects to find and track the area of the maximum pixel density. CamShift works on a principle of an imaginary window that iteratively slides toward the weighted centroid of all the pixels in that window. The size and rotation of the window dynamically adapt in each step. The algorithm eventually converges to a local maximum density area and tracks it as can be seen in Figure 12. In the figure, the green cross represents the centroid of the window found by CamShift. Since the value of the pixels in the backprojection correspond to the probability that given pixel belongs to the object, CamShift finds the group of pixels with the highest local probability of belonging to the object. The centroid of the window is then taken as the position of the object, **x**.

### 3.2. Shape Analysis for Orientation Estimation

The orientation estimation is based on the analysis of the shape of the object's blob. It is assumed that the major axis of the object's blob indicates the object's blob heading. This assumption is reasonable for USVs as they usually have the major axis going from stern to bow facing the direction of movement. Four techniques for estimation of the principal axis of a blob were examined: line fitting, rectangle fitting, principal component analysis (PCA), and ellipse fitting. The ellipse fitting had the lowest orientation estimation error in preliminary experiments and therefore was the only one used in the final experiments presented in section 5.

The first technique was to fit a line through the blob using linear regression. The regression analysis method of least squares was used. It uses the M-estimator method to find the best fitting line by iteratively applying the weighted least-squares algorithm. It finds the line minimizing the sum of the squares of residuals, $\underset{i}{\sum }\frac{r^{2}}{2}$. A residual *r*_*i*_ is defined as the distance between the original point *i* and the point on the fitted line approximating the original point *i*. This technique is more suitable for fitting a line to multiple data points and did not work well for a blob, where all the points are immediately adjacent to each other.

The second technique was to fit a rectangle with the minimum possible area that would still enclose the entire blob. An algorithm proposed in Freeman and Shapira (1975) was used. This algorithm first determines the minimal-perimeter convex polygon enclosing the points (i.e., convex hull). Then, it selects the minimum area rectangle containing this polygon. The idea was that the rectangle's major axis should correspond to the object's major axis. Unfortunately, this technique was not very accurate. The rectangle would sometimes fit in a way that the object would be enclosed diagonally, so the orientation would be estimated incorrectly.

The third technique was principal component analysis taking the principal component as the major axis (Abdi and Williams, 2010). This technique finds the eigenvectors that are the principal components of data points. The eigenvector with the highest eigenvalue represents the major axis of the data. However, this technique suffered from a similar problem as line fitting. It is more suitable for multiple data points, but it did not work well for a single blob where all the points are immediately adjacent to each other.

The fourth technique was to fit an ellipse with the minimum possible area that would still enclose the entire blob. The algebraic distance algorithm was used (Fitzgibbon and Fisher, 1995). This algorithm finds an ellipse enclosing the points while minimizing the least-squares of distances of the points to the ellipse. The major axis of the ellipse then approximates the major axis of the blob. Unlike the rectangle fitting, ellipse does not have the extra corners preventing the blob to fit diagonally. Therefore, this approach worked better than fitting a rectangle and overall the best from the four examined techniques. An example of the fitted ellipse and the estimated orientation can be seen in Figure 12. The green ellipse represents the ellipse fitted to the blob and the yellow line is the major axis of this ellipse approximating the blob orientation.

## 4. Implementation

The proposed approach was implemented to validate the feasibility and to compare the two algorithms for position estimation. Physical platforms used for implementation were a USV (EMILY), a UAS (either DJI Phantom 3 Professional or DJI Inspire 1), or a visual camera as a UAS substitute (GoPro HERO4 Black). The software was implemented on a macOS laptop computer in C++ using OpenCV library. The input was either prerecorded video files or UAS live video stream.

The physical robot platforms used for implementation were Hydronalix Emergency Integrated Lifesaving Lanyard (EMILY) as the USV, and DJI Phantom 3 Professional or DJI Inspire 1 as the UAS, or a GoPro HERO4 Black camera as a substitution for UAS. EMILY is a fast rescue USV covered with a red flotation device. It has maximum speed of 22 m s^−1^ and size of 120 cm length and 28 cm beam. It is designed to move through high surf, currents, and swift water. EMILY can be seen in Figure 1. DJI Phantom 3 Professional is a small (1.3 kg) inexpensive ($800) quad-rotor UAS with 23 min flight time. It is equipped with a gimbaled visual camera that provides up to 4K resolution with 94° field of view and 20 mm focal length. The video is streamed over 2.4 GHz downlink to a ground controller in the resolution of 720p at 30 frames per second (fps) with 220 ms latency. The controller can be equipped with optional DJI HDMI Output Module to provide mini-HDMI video output. DJI Inspire 1 is a small (2.9 kg) inexpensive ($2000) quad-rotor UAS with 18 min flight time. It is also equipped with a gimbaled visual camera that provides up to 4K resolution with 94° field of view and 20 mm focal length. The video is streamed over 2.4 GHz downlink to a ground controller in the resolution of 720p at 30 frames per second (fps) with 220 ms latency. The controller is equipped with mini-HDMI video output by default. DJI Inspire 1 can be seen in Figure 1. GoPro HERO4 Black is a non-gimbaled visual camera that provides up to 4 K resolution (at 30 fps) or up to 240 fps (at 720p resolution) with 65–123° field of view and 17–35 mm focal length. The video recordings are saved on a Micro SD card or can be streamed over 2.4 GHz Wi-Fi.

The software was implemented in *C++* using *OpenCV* library and executed on a macOS laptop computer. The algorithms presented in section 3 were implemented using the following OpenCV functions with default parametrization except where specified otherwise: The Gaussian blur was implemented using GaussianBlur function and kernel size was 21 × 21 px. The conversion from RGB to HSV color space was implemented using cvtColor function. The value histogram equalization used equalizeHist function. For the thresholding algorithm, the thresholds were computed using inRange function, the erosion used erode function with kernel size 2 × 2 px and was applied twice in a row, the dilation used dilate function with kernel size 16 × 16 px and was applied twice in a row, and contour detection used findContours function with simple chain approximation mode (compressing horizontal, vertical, and diagonal segments and leaving only their endpoints). For the histogramming algorithm, the histogram was calculated using calcHist function with histogram size 16, hue histogram backprojection was computed using calcBackProject function, and CamShift was applied using CamShift function with termination criterion either 10 iterations or desired accuracy equal to 1, whichever came first. For orientation estimation algorithms, line fitting used fitLine function implementing the least-squares method as a distance in the M-estimator with the default values for sufficient accuracy, rectangle fitting used minAreaRect function, the principal component analysis was done using PCA class, and ellipse fitting used fitEllipse function. For both thresholding and histogramming algorithms, HSV, and saturation and value thresholds, respectively, were not fixed and were tuned for each trial using implemented graphical user interface (GUI). The GUI was also used to get user input for the construction of the hue histogram in the case of the histogramming algorithm. The code was executed a macOS laptop computer. The hardware configuration of the laptop was the following: processor 2.5 GHz Intel Core i7-4870HQ (Turbo up to 3.7 GHz), memory 16 GB 1.600 MHz DDR3, and no graphical processing unit acceleration.

The input was either video files recorded by UAS/camera onboard or live video feed streamed from UAS. The prerecorded input files were in either MP4 or MOV format. The live video feed stream was used once with DJI Phantom 3 Professional. Since this UAS was not equipped with the optional DJI HDMI Output Module, the video feed had to be streamed from a control tablet (Samsung Galaxy Tab S) connected via USB to the UAS controller. An Android application Screen Stream Mirroring was used to stream the screen of the tablet over the Internet using Real Time Streaming Protocol (RTSP). The stream parameters were set to resolution of 640p at 30 fps, 2048 kbit s^−1^ bit rate, and H.264 encoding.

## 5. Experiments

The goals of the experiments were to validate the feasibility of the proposed approach to visual pose estimation and to compare the thresholding and histogramming algorithms. Four physical trials were performed to measure the position and orientation estimation error of the thresholding and histogramming algorithms: Trial 1 in an outdoor environment using moving camera and an extremely oblique view angle 85°, Trial 2 in an outdoor environment with moving UAS flying at lower altitude (5–15 m), Trial 3 in an outdoor environment with static UAS flying at higher altitude (30 m), and Trial 4 in indoor environment testing robustness under different viewpoints and live video stream input. A position and orientation error relative to ground truth were computed by comparing the position and orientation output with manually annotated ground truth position and orientation.

### 5.1. Experimental Methodology

The experiments tested the following hypothesis. *H*: The position and orientation estimation error for histogramming will be lower than the position and orientation estimation error for thresholding when compared to manually annotated ground truth. The materials used in the experiments were a USV (EMILY), a UAS (DJI Phantom 3 Professional, DJI Inspire 1, or GoPro HERO 4 Black as a substitution for UAS), and computing hardware (a laptop computer). Four physical trials were performed and the details about those trials are summarized in Table 2. The two metrics were a position and orientation estimation error relative to manually annotated ground truth.

**Table 2:** The trials varied in physical configuration and environmental conditions as can be seen from the parameters of the trials.

	**Trial 1**	**Trial 2**	**Trial 3**	**Trial 4**
Date	03/28/2016	04/23/2016	05/10/2016	07/05/2016
Time	16:30	15:30	11:00	13:00
Location	Lake Bryan, Bryan, Texas	Fort Bend County, Texas	Lake Bryan, Bryan, Texas	College Station, Texas
Outdoors	✓	✓	✓	✕
Water type	Lake	Flood	Lake	N/A
USV	Hydronalix EMILY	Hydronalix EMILY	Hydronalix EMILY	Hydronalix EMILY
USV control method	Teleoperation	Teleoperation	GPS Waypoints	N/A
USV speed	Up to 4 m s^−1^	Up to 5 m s^−1^	Up to 2 m s^−1^	0 m s^−1^
UAS/Camera	GoPro HERO4 Black	DJI Inspire 1	DJI Phantom 3 Professional	DJI Phantom 3 Professional
Video streaming	✕	✕	✕	✓
UAS speed	0 m s^−1^	Up to 9 m s^−1^	0 m s^−1^	Up to 1 m s^−1^
Altitude (*a*)	2 m	5 to 15 m	30 m	0 to 1 m
View angle (α)	Up to 85°	Up to 78°	Up to 73°	Up to 90°
Distance UAS to USV	Up to 23 m	Up to 70 m	Up to 100 m	Up to 7 m
Cloud cover	Clear	Clear	Mostly Cloudy	N/A
Wind	ENE 13 km h^−1^	N 7 km h^−1^	S 15 km h^−1^	N/A
Temperature	23 °C	27 °C	28 °C	23 °C
Precipitation	✕	✕	✕	N/A

*Green tick indicates the study was outdoors and red cross indicates the trial was not outdoors. For video streaming, it indicates if the trial used video streaming. For precipitation, it indicates if the trial had any precipitation*.

Trial 1 was an outdoor trial in a lake intended to test an extremely oblique view angle (85°) and used elevated (2 m) ground-based camera instead of a UAS. The GoPro HERO4 Black camera was handheld and moved to keep the USV in the field of view, therefore the video was shaking and moving. The USV was teleoperated at a speed of up to 4 m s^−1^ in a way to frequently change its distance (up to 23 m) and orientation relative to the camera. The trial was performed late in the afternoon (16:30) with the sun being low over the horizon. The camera was facing in the west general direction so the sun was sometimes in the field of view of the camera causing challenges with the white balance of the resulting video. The sky was clear so there were no effects from clouds. The view from the camera looking at the USV during this trial can be seen in Figures 4–7.

Trial 2 was an outdoor trial that used a low flying (5–15 m), fast moving (up to 9 m s^−1^) UAS frequently changing viewpoints of a teleoperated USV. Both the USV and the UAS moved with frequent changes in direction and speed (USV speed was up to 5 m s^−1^). The UAS was sometimes following the USV and changed viewpoints frequently so the USV was viewed from different sides. The distance between the UAS and USV change frequently being up to 70 m. The view angle from nadir also changed frequently being up to 78°. The relative position of the UAS and the sun was also changing causing the USV to be visible from both the sun-illuminated side and non-illuminated side. The sky was clear so there were no effects from clouds. This trial was done during Fort Bend County, TX, 2016 floods so the USV was operated in flood water. The trial in progress can be seen in Figure 1.

Trial 3 was an outdoor trial in a lake that used a high flying (30 m), stationary UAS flying over the shore and looking at the USV at an oblique view angle of up to 73°. The UAS was flying directly above the shore while the USV was operated in GPS waypoints mode at speeds of up to 2 m s^−1^ far away in the water causing the distance between the UAS and the USV to be large (up to 100 m) and the view angle to be oblique (up to 73°). The cloudy weather caused the USV to transition from sun-illuminated areas to shadows and back frequently. The site for the experiment was the same as during Trial 1 with the camera facing in the same general direction, however, this time the trial was done in the late morning (11:00) causing different illumination. The view from the UAS looking at the USV during this trial can be seen in Figure 2.

Trial 4 was performed in an indoor environment testing the robustness under different viewpoints and using UAS live video stream as input. There was a constant illumination from ceiling fluorescent lamps. The USV was stationary, and the UAS was carried around at speed up to 1 m s^−1^ to observe the USV from different angles and distances (up to 7 m). Because the UAS was carried, it was shaking and moving. The USV was placed on an elevated platform so the altitude difference between the USV and UAS was from 0 to 1 m causing the view angle to be up to 90°. The input video was streamed live at the resolution of 640p at 30 fps with 2048 kbit s^−1^ bit rate from the control tablet of the UAS using RTSP (as described in section 4) to test the performance of the algorithms on a live video stream input. Since a USV might look different from different view angles, this trial tested if the algorithms work correctly for all the possible viewpoints.

The methodology for each trial was to capture the USV in operation using UAS/camera, take the video as input, adjust parameters to local conditions, run both algorithms (thresholding and histogramming), and compare the estimated pose with ground truth. In each trial, a UAS/camera was used to capture a video of a USV in operation. The video was either recorded onboard UAS/camera (Trial 1, Trial 2, and Trial 3) or live streamed (Trial 4). Before executing the algorithms, the parameters (HSV thresholds for thresholding, and hue and saturation thresholds for histogramming) were adjusted to local conditions for each trial. Then, both algorithms for position estimation (thresholding and histogramming) with ellipse fitting algorithm for orientation estimation were executed on the video frames to get the estimated pose. This estimated pose was then compared with manually annotated ground truth pose to compute the error.

The metric used for comparison of the estimated pose with ground truth was composed from two parts, position estimation error and orientation estimation error. Both the position estimation error and the orientation estimation error were computed relative to manually annotated ground truth. The video frames were manually annotated for ground truth USV's blob centroid position **x**_*g*_(*t*) and ground truth USV's blob orientation (i.e., heading) θ_*g*_(*t*) both in {*I*} for frame *F*(*t*), ∀*t* ∈ [1..*n*]. The particular algorithm's output was the estimated position of USV's blob centroid **x**_*o*_(*t*) and the estimated orientation of USV's blob θ_*o*_(*t*) in {*I*} for frame *F*(*t*), ∀*t* ∈ [1..*n*]. Then for a frame *F*(*t*), the position estimation error was computed as *e*_**x**_(*t*) = ‖**x**_*g*_(*t*) − **x**_*o*_(*t*)‖_2_ and the orientation estimation error was computed as $e_{\theta }\left(t\right)=\frac{\pi }{2}-\theta_{g}\left(t\right)-\theta_{o}\left(t\right)-\frac{\pi }{2}$. The final metric was mean, median, and standard deviation of *e*_**x**_(*t*) and *e*_θ_(*t*) for *t* ∈ [1..*n*].

### 5.2. Results

While histogramming had statistically significantly lower position estimation error compared to thresholding for all four trials (*p*-value ranged from ~0 to 8.23263 × 10^−29^), it only had statistically significantly lower orientation estimation error for Trial 1 and Trial 3 (*p*-values 3.51852 × 10^−39^ and 1.32762 × 10^−46^, respectively). The mean position estimation error for histogramming was quite low ranging from 7 px to 43 px. The mean orientation estimation error, on the other hand, was quite large being in the best case 0.134 rad (histogramming during Trial 1) and in the worst case even 0.480 rad (thresholding during Trial 3). Table 3 summarizes mean, median, and standard deviation of the position estimation error (*e*_**x**_) and orientation estimation error (*e*_θ_) for both histogramming and thresholding for all four trials.

**Table 3:** The results of the four experiments listing the position estimation error, the orientation estimation error, and the update rate for both thresholding and histogramming algorithms per each trial.

		**Trial 1**	**Trial 2**	**Trial 3**	**Trial 4**
Location		Lake Bryan, Bryan, Texas	Fort Bend County, Texas	Lake Bryan, Bryan, Texas	Laboratory
Video resolution		1, 920 × 1, 080	1, 920 × 1, 080	2, 132 × 1, 200	1, 024 × 640
Observations		480	5, 934	6, 315	2, 249
**Thresholds**					
Thresholding	Hue	[0, 10]∪[160, 180]	[0, 10]∪[160, 180]	[0, 10]∪[160, 180]	[0, 10]
	Sat.	[58, 255]	[120, 255]	[30, 255]	[167, 255]
	Value	[81, 255]	[100, 255]	[10, 255]	[50, 255]
Histogramming	Sat.	[52, 255]	[120, 255]	[30, 255]	[130, 255]
	Value	[10, 255]	[100, 255]	[10, 255]	[10, 255]
**Position estimation error (e _**x**_)**					
Thresholding	Mean	440 px	15 px	311 px	100 px
	Median	51 px	9 px	10 px	47 px
	SD	434 px	51 px	385 px	126 px
Histogramming	Mean	15 px	8 px	7 px	43 px
	Median	13 px	6 px	6 px	27 px
	SD	9 px	5 px	4 px	44 px
**T**-Value for *H*^**x**^		−21.458303	−11.13177414	−63.23634755	−21.01028455
**P**-Value for *H*^**x**^		2.24164 × 10^−72^	8.23263 × 10^−29^	~0	6.02253 × 10^−92^
**Orientation estimation error (e_θ_)**					
Thresholding	Mean	0.462 rad	0.178 rad	0.480 rad	0.141 rad
	Median	0.243 rad	0.099 rad	0.271 rad	0.037 rad
	SD	0.485 rad	0.260 rad	0.477 rad	0.251 rad
Histogramming	Mean	0.134 rad	0.180 rad	0.365 rad	0.183 rad
	Median	0.076 rad	0.119 rad	0.147 rad	0.044 rad
	SD	0.163 rad	0.202 rad	0.427 rad	0.325 rad
*T*-value for *H*^θ^		−14.055826	0.515911	−14.345444	4.854452
*P*-value for *H*^θ^		3.51852 × 10^−39^	6.97037 × 10^−1^	1.32762 × 10^−46^	9.99999 × 10^−1^
**Update rate**					
Thresholding		5 Hz	6 Hz	2 Hz	11 Hz
Histogramming		7 Hz	8 Hz	3 Hz	14 Hz

*Both the position estimation error and orientation estimation error are listed in terms of mean, median, and standard deviation, and t-value and p-value for hypothesis H^x^ and H^θ^, respectively. The table also contains location of each trial, resolution of the input video, number of frames in the video (observations), and parameters (thresholds) of each algorithm that were used for particular trial (Sat. is an abbreviation for saturation). Green and red are used to compare thresholding and histogramming. Red indicates higher error and green indicates lower error. For p-value, green indicates statistical significance of corresponding hypothesis and red otherwise*.

The main hypothesis, *H*, was divided into two hypotheses, *H*^**x**^ for position estimation error metric (*e*_**x**_) and *H*^θ^ for orientation estimation error metric (*e*_θ_), and tested for statistical significance using a *t*-test. The hypothesis for position estimation error was *H*^**x**^: The position estimation error for histogramming ($e_{\text{x}}^{\left(H\right)}$) will be lower than the position estimation error for thresholding ($e_{\text{x}}^{\left(T\right)}$). The hypothesis for orientation estimation error was *H*^θ^: The orientation estimation error for histogramming ($e_{\theta }^{\left(H\right)}$) will be lower than the orientation estimation error for thresholding ($e_{\theta }^{\left(T\right)}$). First, two-sample two-tailed *f*-test for equal variances with 5 % significance level indicated unequal variances for all the cases. Then, two-sample one-tailed (left) *t*-test assuming unequal variances (using Satterthwaite's approximation) was used to test the two hypothesis for all four trials leading to a total of eight t-tests. The *t*-values were computed as follows:

$$t=\frac{\bar{A}-\bar{B}}{\sqrt{\frac{s_{A}^{2}}{N_{A}}+\frac{s_{A}^{2}}{N_{A}}}}$$

Where $\bar{A}$ and $\bar{B}$ are the sample means, *s*_*A*_ and *s*_*B*_ are the sample standard deviations, and *N*_*A*_ and *N*_*B*_ are the sample sizes. Table 3 summarizes *t*-values and *p*-values for each hypothesis *H*^**x**^ and *H*^θ^ for all four trials.

The *t*-test confirmed the statistical significance of the hypothesis *H*^**x**^ for position estimation error metric (*e*_**x**_) on 5 % significance level for all four trials meaning histogramming had statistically significantly lower position estimation error (*e*_**x**_) compared to thresholding for all four trials. The null hypothesis was $H_{0}^{\text{x}}:\bar{e_{\text{x}}^{\left(H\right)}}=\bar{e_{\text{x}}^{\left(T\right)}}$ and the alternate hypothesis was $H_{a}^{\text{x}}:\bar{e_{\text{x}}^{\left(H\right)}}<\bar{e_{\text{x}}^{\left(T\right)}}$, where $\bar{e_{\text{x}}^{\left(H\right)}}$ and $\bar{e_{\text{x}}^{\left(T\right)}}$ were mean position estimation error of histogramming and thresholding, respectively. The resulting *p*-values were 2.24164 × 10^−72^, 8.23263 × 10^−29^, ~0, 6.02253 × 10^−92^ for Trials 1–4, respectively.

The *t*-test confirmed the statistical significance of the hypothesis *H*^θ^ for orientation estimation error metric (*e*_θ_) on 5 % significance level for Trial 1 and Trial 3, but failed to confirm the statistical significance of the hypothesis for Trial 2 and Trial 4. Therefore, histogramming had statistically significantly lower mean orientation estimation error (*e*_θ_) compared to thresholding for Trial 1 and Trial 3, but the results for Trial 2 and Trial 4 were inconclusive. The null hypothesis was $H_{0}^{\theta }:\bar{e_{\theta }^{\left(H\right)}}=\bar{e_{\theta }^{\left(T\right)}}$ and the alternate hypothesis $H_{a}^{\theta }:\bar{e_{\theta }^{\left(H\right)}}<\bar{e_{\theta }^{\left(T\right)}}$, where $\bar{e_{\theta }^{\left(H\right)}}$ and $\bar{e_{\theta }^{\left(T\right)}}$ were mean orientation estimation error of histogramming and thresholding, respectively. The resulting *p*-values were 3.51852 × 10^−39^, 6.97037 × 10^−1^, 1.32762 × 10^−46^, and 9.99999 × 10^−1^ for Trials 1–4, respectively.

The histogramming had a lower mean, median, and standard deviation of the position estimation error (*e*_**x**_) compared with thresholding. The mean of *e*_**x**_ for histogramming was quite low ranging from 7 to 43 px with median ranging from 6 to 27 px. On the other hand, the mean position estimation error for thresholding was relatively high ranging 15 to 440 px with median ranging from 9 to 51 px. The histogramming had much lower standard deviation of *e*_**x**_ (4 to 44 px) compared to thresholding (51 to 434 px).

The mean orientation estimation error (*e*_θ_) was relatively high for both algorithms ranging from 0.134 rad for histogramming in Trial 1 to 0.480 rad for thresholding in Trial 3. The median of *e*_θ_ was ranging from 0.037 rad for thresholding in Trial 4 to 0.271 rad for thresholding in Trial 3. The standard deviation was also relatively large ranging from 0.163 rad for histogramming in Trial 1 to 0.485 rad for thresholding in Trial 1.

The update rate was higher for histogramming than thresholding for all the Trials. The update rate value was dependent on the video resolution. It was highest for Trial 4 (resolution 1, 024 × 640) being 11 Hz for thresholding and 14 Hz for histogramming. It was lowest for Trial 3 (resolution 2, 132 × 1, 200) being 2 Hz for thresholding and 3 Hz for histogramming.

## 6. Discussion

The histogramming algorithm had statistically significantly lower position estimation error than thresholding when compared with manually annotated ground truth (*p*-value ranged from ~0 to 8.23263 × 10^−29^), showed the feasibility to variations in environmental conditions and physical settings in three outdoor trials and one indoor trial, and required one less parameter than thresholding. There were, however, three problems. The orientation estimation error was quite large for both algorithms (0.134 to 0.480 rad), both algorithms required manual tuning, and both algorithms were not robust enough to recover from significant changes in illumination conditions. The experiments could have been improved to measure the robustness of the algorithms in addition to their precision by introducing a metric counting the number of times particular algorithm completely lost track of the object. Future work will focus on adapting machine learning methods used for 6D pose estimation to address the issues with the robustness and the need for manual tuning.

### 6.1. Histogramming Algorithm Is Better Than Thresholding for Position Estimation

The histogramming algorithm had a lower position estimation error than thresholding due to its capability to create the object's model in local illumination conditions and to capture more information about the hue distribution of the object. It showed the feasibility to varying configurations and environmental conditions in physical trials. It also required less parameter tuning than thresholding and had a higher update rate.

The histogramming algorithm had statistically significantly lower position estimation error than thresholding when compared with manually annotated ground truth for two reasons: it uses a hue model of the object created in local illumination conditions and it can capture more information about the hue distribution of the object than thresholding. First, the histogramming algorithm creates a hue histogram model of the object in local illumination conditions. In the case of the thresholding algorithm, the hue threshold has to be set manually and is not computed from the actual appearance of the object in specific illumination conditions. There are four environmental conditions that can change the appearance of hue: time of day, time of year, weather, and surrounding environment colors (water and the surrounding environment all reflect light onto the object). Second, a hue histogram can capture more information about the hue properties of the object compared to thresholding. The thresholding algorithm only models the hue of the object as a single range of acceptable hue values. On the other hand, the histogramming algorithm works with the entire histogram of hue specifying a hue distribution of the object of interest.

The histogramming algorithm showed feasibility for varying configurations and environmental conditions in physical trials. Despite the variations in trials, the mean position estimation error for histogramming was still relatively low ranging from 7 to 43 px. The three outdoor field trials were performed at three times of year (March, April, May), three times of day (11:00, 15:30, 16:30), in two water types (lake and flood water), using two control modes of the USV (teleoperation and GPS waypoints), with USV speed ranging from 2 to 5 ms^−1^, with three UASs/camera platforms (DJI Phantom 3 Professional, DJI Inspire 1, GoPro HERO4 Black), with UAS speeds of up to 9 m s^−1^, at altitudes ranging from 2 to 30 m, at view angles ranging from 73 to 85°, at distance between UAS/camera and UAS of up to 100 m, with two different weather conditions (clear and mostly cloudy sky), at three different relative positions of UASs/cameras (an extremely oblique view from static camera yawing to follow the USV, UAS following the USV, stable UAS above shore), and with both the sun-illuminated side and non-illuminated side of the USV being visible. In addition to the three outdoor trials, one indoor trial tested the position estimation from different viewpoints with the view angle of up to 90° from nadir.

The histogramming algorithm had one less parameter than thresholding and therefore required less manual tuning. The thresholding required to manually set all three HSV thresholds. The histogramming algorithm only required to set saturation and value thresholds.

The histogramming algorithm had a higher update rate than thresholding in all the trials. This indicates a higher efficiency of the histogramming algorithm compared to thresholding. Nevertheless, the update rate for both algorithms was enough to enable visual navigation.

### 6.2. Limitations of Proposed Method

A total of three problems with the proposed method were identified. First, the orientation error was quite high due to the oblique view angle. Second, both algorithms required manual tuning before each mission. Third, the algorithms were not robust to significant illumination changes.

The orientation estimation error was quite large for both thresholding and histogramming ranging from 0.134 to 0.480 rad because of perspective distortion caused by the oblique view. Because of the oblique view angle, the shape of the object is not constant and may vary depending on the relative pose of the object to the camera. In the extreme case (object faces the camera and is it is far away), even a cylindrical object might have a circular profile having no significant major axis. This is illustrated in Figure 2 where a USV's blob is circular making it hard to estimate the orientation by shape analysis. For this reason, an inverse perspective warping will probably be necessary to reduce the perspective effects introduced with an oblique view angle.

Both thresholding and histogramming required manual tuning for local conditions requiring to adjust three and two parameters, respectively. The thresholding required setting HSV thresholds and histogramming required setting saturation and value thresholds and selecting the object before the visual pose estimation could start. If those parameters were set incorrectly, it would likely lead to a complete failure. For example, if the lower saturation threshold was set higher than the object's saturation, the object would be filtered out. On the other hand, if the lower saturation threshold was set too low, it would fail to filter out parts of the environment with similar hue, but lower saturation.

While histogramming algorithm had relatively low position estimation error (mean ranging from 7 to 43 px), when it loses track of the object due to significant illumination changes, it is very unlikely to recover by itself and human input is required to reselect the object to rebuild the histogram model. The reason is that the algorithm cannot adapt to significant changes in illumination conditions during a single mission. The illumination conditions may change very abruptly with changes in the relative position of all UAS, the object, and sun. For example, the camera may face the sun distorting white balance in the entire view as can be seen in Figure 5. This causes the object's color to be very similar to the color of water violating the assumption that the object's color is unique in the environment. Another example is presented in Figure 4 illustrating how significantly can illumination conditions change during a single run. A method based on machine learning might lead to better robustness to illumination changes and might require less manual tuning before each mission.

### 6.3. Improving Experiments and Machine Learning for Future Work

The experiments could have been improved by introducing an additional metric that would reflect robustness to changing illumination conditions. Additionally, not all the experiments used UAS and not all were outdoors as one trial used a camera instead of UAS and one trial was indoors. The future work will focus on exploring machine learning methods to increase robustness to changing illumination conditions and reduce the need for manual parameter tuning.

An additional metric could have been introduced to test for robustness to changing illumination by reflecting how many times did a particular algorithm completely fail and required a manual change of parameters to recover. Since the parameters of the algorithms were tuned before each trial, the results reflected the precision of the algorithms rather than their general robustness to changing conditions. To test for robustness, each experiment would be started with default parameters and the metric would count how many times those parameters had to be changed to keep the track of the object.

The experiments could have been improved by using a UAS in all the trials and by performing all the trials outdoors. Only two of the four trials were performed outdoors with a UAS and moving USV. Trial 1, while outdoors, used a visual camera instead of UAS. However, this provided a good challenge since the view angle was extremely oblique (about 85°) due to very low altitude (2 m). The camera was not stabilized in any way and was hand-held and moving to keep the USV in the field of view. Trial 4 was performed indoors and the USV was not moving. However, this enabled to cover different viewpoints around the USV. The UAS was carried around the USV in the way to cover all the possible view angles.

The aim of future work will be to explore machine learning methods for visual pose estimation with a goal to increase robustness to illumination changes and eliminate the need for manual tuning. There is a large body of work on visual tracking of objects, however, those methods generally do not estimate orientation. There is very active research on visual 6D pose estimation of objects, however, those methods are usually used for robotic manipulation and are done indoors at a very short distance (e.g., objects placed on a table and camera on a manipulator). Most of those methods use depth information from RGB-D cameras, but some use only monocular RGB cameras. The state of the art methods using monocular RGB cameras are Do et al. (2018), Li et al. (2018), Tremblay et al. (2018). The goal of the future work will be to explore if those methods can be adapted to the problem in hand. Machine learning methods might improve robustness while eliminating the need for manual parameter tuning.

## 7. Summary

This article focused on the problem of visual pose estimation of USV from UAS with the following requirements:

- The operating environment is an outdoor water environment.
- Both UAS and USVs are moving (up to 22 and 10 m s^−1^, respectively).
- The UAS is looking at the scene at an oblique view angle (up to 85° from nadir) from large distance (up to 180 m).
- The output has to be full pose (both position and orientation) provided in real-time (more than 5 Hz).

The presented approach was based on the object's hue invariance. Two algorithms for position estimation were presented: thresholding and histogramming. Shape analysis was used for orientation estimation assuming the major axis of the object's blob indicates the object's blob heading. Four techniques for estimation of the principal axis of a blob were examined: line fitting, rectangle fitting, principal component analysis (PCA), and ellipse fitting.

Four physical experiments were performed to validate the feasibility of the proposed approach for visual pose estimation and to compare the thresholding and histogramming algorithms. A position and orientation error relative to ground truth were computed.

The histogramming algorithm had a lower position estimation error, showed feasibility for varying environmental conditions and physical settings, and required fewer parameters than thresholding. However, three problems were identified. The orientation estimation error was quite large for both algorithms, both algorithms required manual tuning, and both algorithms were not robust enough to recover from significant changes in illumination conditions.

To conclude, the histogramming algorithm with ellipse fitting for orientation estimation should be good enough if enhanced with an inverse perspective warping to reduce the orientation estimation error and if no significant changes in illumination are expected as demonstrated by the low position estimation error (7 to 43 px). If there are significant lighting changes, manual input might be necessary to recreate the hue histogram. To increase the robustness to significant lighting changes without the necessity to recreate the hue histogram, a machine learning approach might ultimately be a better solution.

## Author Contributions

JD is responsible for the literature review, approach, implementation, experiments, and writing this manuscript. RM is the principal investigator for the project and provided guidance in all the parts and revised each draft of this manuscript.

## Conflict of Interest Statement

The authors declare that the research was conducted in the absence of any commercial or financial relationships that could be construed as a potential conflict of interest.
